# Regulatory roles of five key USP family deubiquitinases in cancer: from mechanisms to targeted therapy advances

**DOI:** 10.3389/fphar.2026.1933967

**Published:** 2026-09-10

**Authors:** Yanan Wang, Jing Shi

**Affiliations:** Department of Pharmacy, Shandong Cancer Hospital and Institute, Shandong First Medical University and Shandong Academy of Medical Sciences, Jinan, China

**Keywords:** cancer, deubiquitinating enzymes, selective inhibitors, targeted therapy, tumor microenvironment, ubiquitin-specific proteases

## Abstract

Ubiquitination is a crucial post-translational modification of proteins in eukaryotic cells. Deubiquitinating enzymes (DUBs) remove ubiquitin molecules from substrate proteins, thereby reversing ubiquitination and maintaining intracellular ubiquitin homeostasis. Dysregulation or dysfunction of DUBs is closely associated with various diseases. Among them, the ubiquitin-specific protease (USP) family, the largest subfamily of DUBs, plays a key regulatory role in tumor initiation and progression. This article systematically reviews the research progress on five representative USP family members closely linked to tumors, including USP7, USP22, USP10, USP35, and USP4, with a focus on their functional mechanisms in regulating major tumor-related signaling molecules and pathways, including p53, PTEN, c-Myc, and PD-L1. It also highlights their dual regulatory roles in tumor proliferation, resistance to apoptosis, metastasis, and immune evasion. Furthermore, this review summarizes the structural basis of USP catalysis and selectivity, the determinants of context-dependent USP functions, and the emerging roles of DUBs in tumor microenvironment remodeling and therapy resistance. We also discuss the latest advances in the development of selective inhibitors and new therapeutic modalities targeting these USPs, including PROTAC-mediated degradation, DUBTAC-mediated tumor suppressor stabilization, and molecular glue strategies. Although targeting DUBs for cancer therapy faces challenges such as substrate diversity, context-dependent functions, and functional redundancy within the family, it remains a promising strategy for tumor treatment. This review provides a theoretical foundation and research directions for further understanding the roles and mechanisms of the USP family in cancer and for developing targeted DUB-based anti-tumor therapies.

## 1 Introduction

Ubiquitination is a reversible post-translational modification that enables precise regulation of protein stability, localization, interactions, and functions through the covalent attachment of a 76-amino-acid ubiquitin molecule to substrate proteins. This process is catalyzed by a sequential enzymatic cascade involving E1 ubiquitin-activating enzymes, E2 ubiquitin-conjugating enzymes, and E3 ubiquitin ligases, with the E3 ligase determining the specificity of substrate recognition (Kim et al., 2026; Komander and Rape, 2012; Yau and Rape, 2016). Ubiquitin molecules can form various types of polyubiquitin chains via their seven lysine residues (K6, K11, K27, K29, K33, K48, K63) or the N-terminal methionine. Different chain types direct distinct fates of the substrate: K48-linked chains primarily mediate proteasomal degradation of substrates, whereas K63-linked and M1 (linear) chains are involved in non-proteolytic functions such as signal transduction, DNA damage repair, and immune responses. The ubiquitin-proteasome system (UPS) is responsible for approximately 80%–90% of intracellular protein degradation, serving as a central mechanism for maintaining protein homeostasis. Dysregulation of ubiquitination can lead to abnormal accumulation or excessive degradation of substrate proteins, which is closely associated with the onset and progression of various major diseases, including cancers, neurodegenerative disorders, cardiovascular diseases, inflammatory conditions, and developmental abnormalities (Yang et al., 2025; Popovic et al., 2014; Swatek and Komander, 2016; Ebadi et al., 2025; Deng et al., 2020).

**FIGURE 1:**
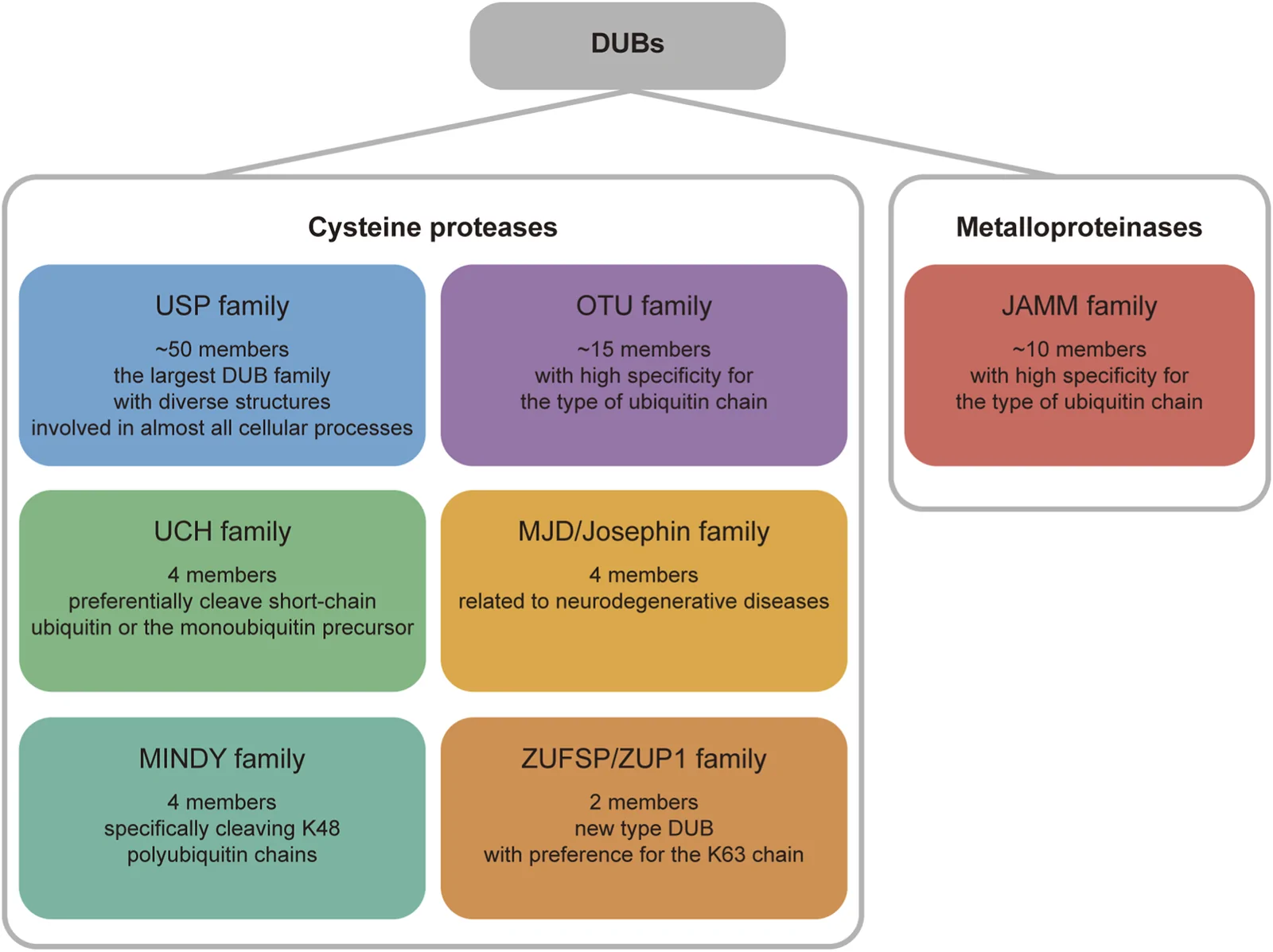
Schematic diagram of deubiquitinating enzyme classification, including cysteine proteases and metalloproteases.

Deubiquitinating enzymes (DUBs) reverse ubiquitination by specifically removing ubiquitin molecules from substrate proteins, thereby maintaining the dynamic balance between ubiquitination and deubiquitination within cells. The human genome encodes approximately 100 DUBs, which are primarily classified into seven families based on differences in their catalytic domains: ubiquitin-specific proteases (USPs), ubiquitin carboxyl-terminal hydrolases (UCHs), ovarian tumor proteases (OTUs), Machado-Joseph disease domain proteases (MJDs), JAMM/MPN domain metalloproteases, zinc finger-containing ubiquitin peptidase 1 (ZUFSP), and motif interacting with ubiquitin (MIU)-containing novel DUBs (MINDYs) (Figure 1) (Snyder and Silva, 2021; Clague et al., 2019; Leznicki and Kulathu, 2017). Among these, the USP family is the largest group of DUBs, comprising more than 50 members with diverse structures and involvement in nearly all cellular processes. In terms of mechanisms of action, USP family members exhibit substrate recognition specificity. Studies have shown that abnormal expression or dysfunction of DUBs is also closely associated with various diseases. In particular, USP family members play critical roles in tumor development, neurodegenerative diseases, and inflammatory responses (Li and Reverter, 2021; Basar et al., 2021; Poondla et al., 2019).

Cancer is a major disease that threatens human life and health. In recent years, a growing number of studies have revealed the relationship between DUBs and tumor development, positioning DUBs as promising therapeutic targets of significant interest (Dewson et al., 2023; Ren et al., 2023; Tang et al., 2025; Harrigan et al., 2018; Lange et al., 2022; Murali et al., 2024; Mennerich et al., 2019). Research has shown that various DUBs are highly expressed in tumor cells and tissues and are associated with poor patient prognosis. Mechanistic studies have further demonstrated that DUBs are involved in regulating multiple cancer-related substrates and downstream signaling pathways, thereby modulating various hallmarks of cancer and the processes of tumor initiation and progression. These include effects on tumor cell proliferation, resistance to cell death (including chemotherapy resistance), tumor invasion and metastasis, and immune escape. In this review, we summarize and discuss five representative USP family members that have been frequently reported and are closely associated with tumors, including USP7, USP22, USP10, USP35, and USP4. Notably, although these five DUBs are representative, they represent only a subset of the numerous members associated with cancer and are by no means exhaustive. We elaborate on their functions in tumor development and the related research progress in detail.

## 2 Structural basis of USP catalytic domains and selectivity determination

The USP family shares a conserved catalytic core composed of approximately 350–400 amino acid residues, whose overall three-dimensional fold exhibits remarkable similarity to that of papain. This domain can be further subdivided into three subdomains, designated as the finger, palm, and thumb regions (Keijzer et al., 2024). The catalytic center is mediated by a Cys-His-Asp/Asn triad, in which the thiol group of the cysteine residue serves as the nucleophile responsible for attacking the isopeptide bond linking the C-terminus of ubiquitin to its substrate, thereby executing the deubiquitination reaction (Shin et al., 2023). Topologically, the finger subdomain primarily engages in recognition and binding of the distal ubiquitin moiety, the palm subdomain accommodates the catalytic triad residues, and the thumb subdomain plays a critical role in ubiquitin recognition and substrate positioning. These three subdomains coordinately construct an intact ubiquitin-binding interface and orient the C-terminal tail of ubiquitin into the catalytic cleft to facilitate efficient hydrolysis (Keijzer et al., 2024).

Precise regulation of USP enzymatic activity relies heavily on conformational rearrangements of the catalytic triad upon ligand binding. In the resting state in the absence of ubiquitin, numerous USP members adopt inactive conformations characterized by misalignment of the catalytic residues, wherein the spatial distance between cysteine and histidine exceeds the geometric threshold permissive for nucleophilic attack. Ubiquitin engagement induces global conformational changes that realign the catalytic triad into a catalytically competent orientation (Pozhidaeva et al., 2017). USP7 represents a paradigmatic example of this regulatory mechanism: binding of ubiquitin to its catalytic domain elicits pronounced conformational transitions, notably including repositioning of the BL2 loop (β-hairpin loop 2), which precisely organizes Cys223, His464, and Asp481 into an active configuration (Gavory et al., 2018). This ligand-induced activation mechanism has also been corroborated in USP15 and USP47, both of which share high sequence homology with USP7 within their catalytic domains (Turnbull et al., 2017).

Despite the high conservation of the overall folding architecture among USP family members, the BL1, BL2, and BL3 loops (β-hairpin loops 1, 2, and 3) located at the rim of the catalytic groove exhibit considerable variations in both sequence length and conformational features. Accumulating evidence indicates that the structural diversity of these loop regions constitutes a key molecular determinant governing substrate specificity and catalytic efficiency across different USP members (Lamberto et al., 2017). For instance, the catalytic cleft of USP7 is relatively broad and shallow, accommodating substrates with diverse structural features. By contrast, although USP47 shares approximately 51% sequence identity with USP7 within the catalytic domain, differences in its BL1 and BL2 loops give rise to markedly distinct substrate preferences (Turnbull et al., 2017). In recent years, structural approaches including X-ray crystallography, cryo-electron microscopy (cryo-EM), and artificial intelligence-assisted structure prediction tools such as AlphaFold have substantially expanded the boundaries of structural biology knowledge regarding the USP family and have furnished robust structural templates for the rational design of specific inhibitors (Song et al., 2025).

The intrinsic conformational plasticity inherent to USP catalytic domains offers multiple avenues for drug development targeting both the active site and allosteric sites. Taking USP7 as an exemplar, the small-molecule inhibitors FT671 and FT827 bind to an inducible dynamic pocket adjacent to the active site, which is situated in close proximity to the ubiquitin-binding interface. Among these, FT671 exerts its inhibitory effect by stabilizing the closed conformation of the catalytic domain, thereby spatially obstructing access of ubiquitin substrates, and demonstrates nanomolar affinity with favorable selectivity over related USP family members (Gavory et al., 2018). Complementarily, allosteric inhibitors exert their modulatory effects by engaging sites distant from the catalytic center. The palm subdomain of USP7 contains a distinctive allosteric binding cavity. Occupancy of this site by small molecules induces conformational alterations that indirectly attenuate catalytic function, without directly competing with ubiquitin for binding to the active site (Song et al., 2025). This allosteric regulatory strategy has been extended to other family members, including USP14 and USP30. Inhibitors developed against these two enzymes specifically recognize structural features surrounding the catalytic cleft and block ubiquitin access to the catalytic channel via steric hindrance, thereby achieving selective inhibition (Lamberto et al., 2017). Collectively, these structural biology investigations into the conformational dynamics and regulatory mechanisms of USP catalytic domains provide a systematic theoretical framework for a deeper understanding of the functional diversity of this family and its therapeutic potential as a drug target.

## 3 Cell context-dependent determinants of USP function

**FIGURE 2:**
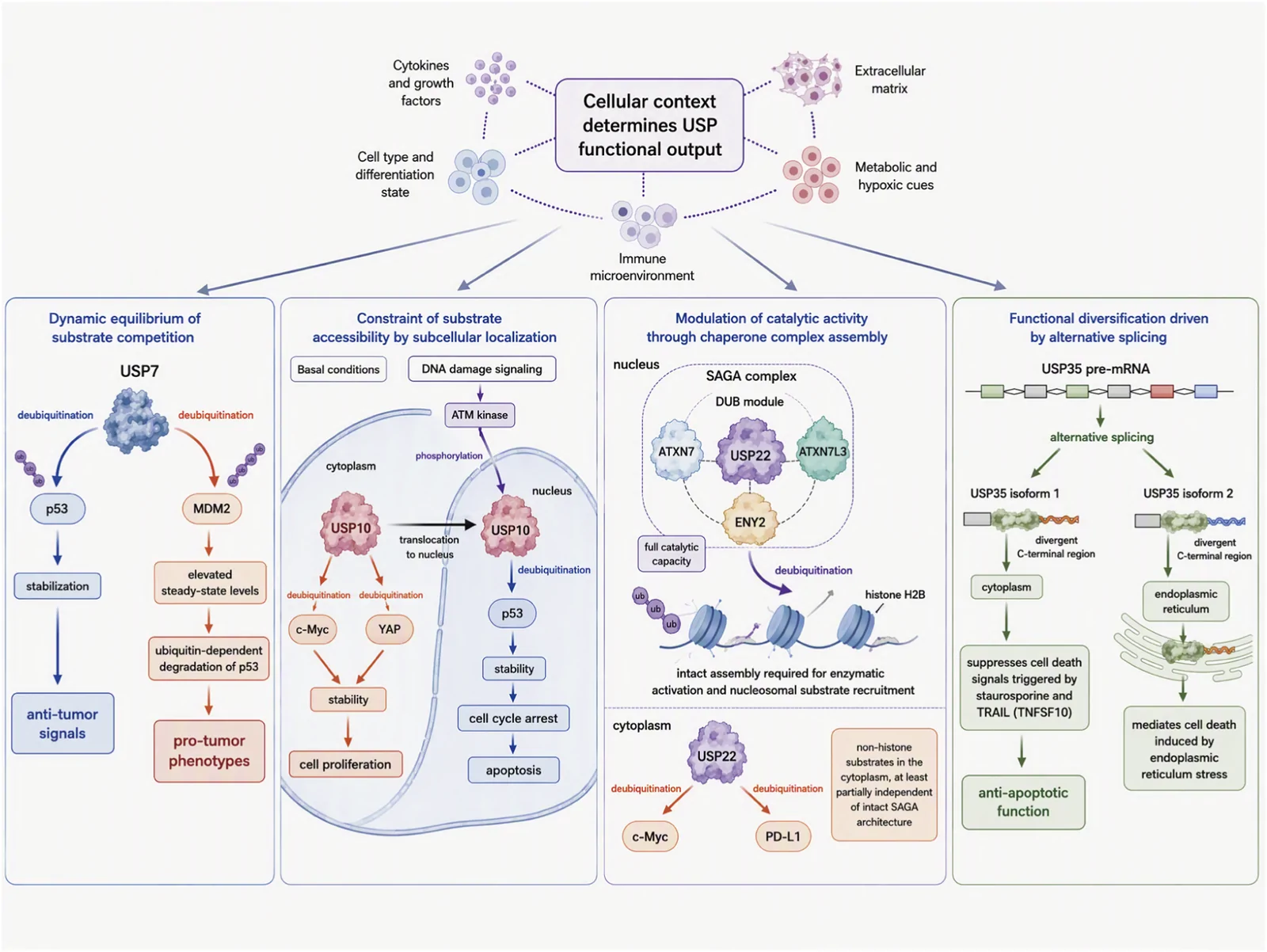
Schematic diagram of determinants of context-dependent USP functions.

USP family members frequently exert mutually antagonistic effects on tumor initiation and progression depending on the cellular context, indicating that their functional outputs are not fixed but rather highly contingent upon dynamic changes in the cellular microenvironment. From a systematic perspective, the following four core dimensions, the dynamic equilibrium of substrate competition, the constraint of substrate accessibility imposed by subcellular localization, the modulation of catalytic activity through chaperone complex assembly, and the functional diversification driven by alternative splicing, collectively constitute a regulatory network that governs the functional orientation of a given USP in specific cellular settings (Figure 2).

### 3.1 Dynamic equilibrium of substrate competition

A single USP molecule typically recognizes and acts upon multiple substrate proteins, stabilizing, through deubiquitination, distinct substrates that possess mutually opposing biological effects, thereby yielding differential or even diametrically opposed net functional outcomes under specific cellular contexts. USP7 serves as a paradigmatic example of this regulatory logic. On one hand, USP7 directly deubiquitinates the tumor suppressor p53 and enhances its protein stability, thereby transducing anti-tumor signals (Gavory et al., 2018). On the other hand, it also deubiquitinates the E3 ubiquitin ligase MDM2 and elevates its steady-state levels, consequently promoting the ubiquitin-dependent degradation of p53 and driving pro-tumor phenotypes. In a given cellular microenvironment, the outcome of competition between these two functional pathways essentially depends on the integrated effects of multiple variables, including the relative abundance of p53 and MDM2, the availability of adaptor proteins, and the post-translational modification landscape of USP7 itself (Gavory et al., 2018).

### 3.2 Constraint of substrate accessibility by subcellular localization

The subcellular compartment in which a DUB resides fundamentally delimits its accessible substrate repertoire and thus constitutes a primary spatial determinant shaping its functional output. USP10 provides a clear illustration of this principle. Under basal conditions, USP10 is predominantly localized in the cytoplasm, where it catalyzes the deubiquitination of oncogenic substrates such as c-Myc and YAP and enhances their stability, thereby driving cell proliferation (Yuan et al., 2010). Upon activation of DNA damage signaling, ATM kinase-mediated phosphorylation of USP10 triggers the translocation of a fraction of the enzyme to the nucleus, where USP10 subsequently targets p53 and enhances its stability, ultimately inducing cell cycle arrest and apoptosis. This phosphorylation event serves as a critical molecular switch that bridges DNA damage stress signals with the functional redirection of USP10.

### 3.3 Modulation of catalytic activity through chaperone complex assembly

A considerable number of USP members must be integrated into multi-subunit protein complexes to achieve full catalytic capacity and acquire appropriate substrate selectivity. USP22, as an integral component of the Spt–Ada–Gcn5 acetyltransferase (SAGA) transcriptional coactivator complex, exhibits deubiquitinating activity toward histone H2B that is strictly dependent on the intact assembly of the DUB module within this complex. Specifically, USP22 is required to form stable protein-protein interaction networks with ATXN7, ATXN7L3, and ENY2, and these interactions are indispensable for both the activation of enzymatic activity and the effective recruitment of nucleosomal substrates (Samara et al., 2010; Köhler et al., 2010). Notably, USP22 is also capable of catalyzing the deubiquitination of non-histone substrates, such as c-Myc and PD-L1, in the cytoplasm, a process that appears to be at least partially independent of the intact SAGA complex architecture. This observation suggests that the assembly state of chaperone complexes may differentially regulate the functional output of USP22 in a substrate class-dependent manner (Morgan et al., 2016).

### 3.4 Functional diversification driven by alternative splicing

Alternative splicing of pre-mRNA generates USP isoforms that differ in domain composition, subcellular distribution, and even functional properties, thereby greatly expanding the regulatory complexity of DUB-mediated signal transduction at the post-transcriptional level. Taking USP35 as an example, this gene produces two isoforms with markedly divergent C-terminal regions through alternative splicing: isoform 1 localizes to the cytoplasm and exerts an anti-apoptotic function by effectively suppressing cell death signals triggered by staurosporine and TNF-related apoptosis-inducing ligand (TRAIL, also known as TNFSF10). In contrast, isoform 2 resides in the endoplasmic reticulum and subsequently mediates cell death induced by endoplasmic reticulum stress (Leznicki et al., 2018). These findings strongly indicate that alternative splicing operates as a functional switch, generating functionally divergent USP variants from a single genetic locus, thereby providing cells with flexible regulatory reserves for responding to diverse physiological or pathological stimuli.

## 4 USP7 and cancer

**FIGURE 3:**
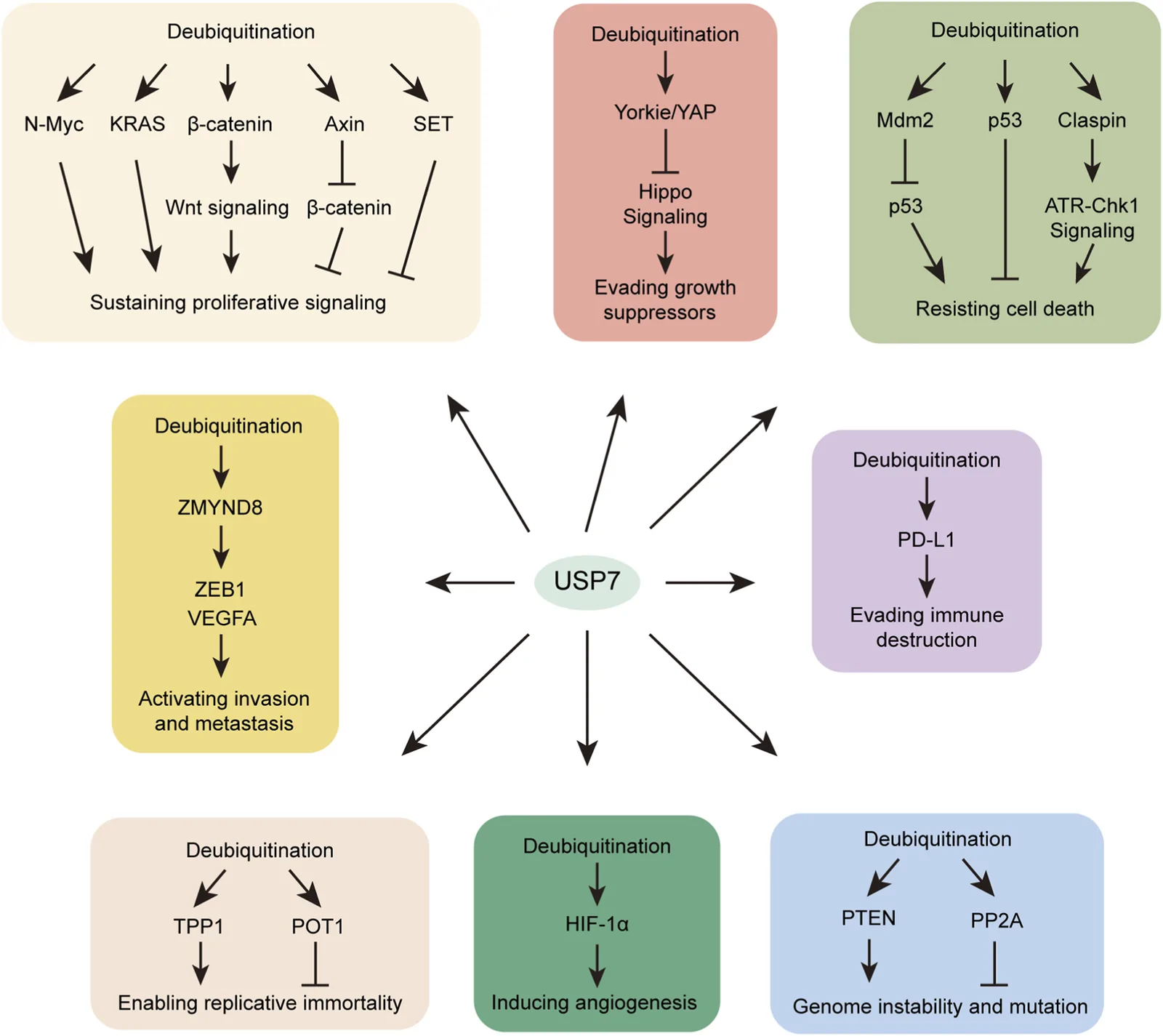
The cancer-related substrates, pathways and cancer hallmarks regulated by USP7. Arrows denote activation or stabilization, bars denote inhibition or degradation.

USP7, also called HAUSP (herpesvirus-associated ubiquitin specific protease), is considered a potential target for tumor therapeutics. Studies have shown that it participates in regulating multiple pathways and processes associated with cancer hallmarks (Hanahan and Weinberg, 2011; Saha et al., 2023; Park and Baek, 2023; Carreira et al., 2023; Zhou et al., 2018). Its regulatory effect on cancer is bimodal and context-dependent, meaning that under certain pathways and conditions, it promotes cancer progression, while under others, it exerts an inhibitory effect (Figure 3; Table 1). Nevertheless, USP7 is currently primarily regarded as an oncogene, given its high expression characteristics observed in various tumor cells.

**TABLE 1:** Substrates, pathways and cancer hallmarks regulated by USP7.

Regulated substrates	Function of mechanism	Tumor promotion (+)/tumor suppression (−)	Cancer hallmarks involved	References
Mdm2	Stabilize Mdm2 and inhibit p53	+	Resisting cell death	(Li et al., 2004)
PTEN	Promote the nuclear export of PTEN and inhibit its function	+	Genome instability and mutation	(Song et al., 2008)
N-Myc	Stabilize N-Myc	+	Sustaining proliferative signaling, resisting cell death	(Tavana et al., 2016; Grunblatt et al., 2020)
KRAS	Stabilize KRAS	+	Sustaining proliferative signaling	(Huang et al., 2024)
β-catenin	Stabilize β-catenin and regulate the Wnt pathway	+	Sustaining proliferative signaling	(An et al., 2017)
p53	Stabilize p53	-	Resisting cell death	(Li et al., 2002; Li et al., 2024b)
Axin	Stabilize Axin and promote the degradation of β-catenin	-	Sustaining proliferative signaling	(Ji et al., 2019)
PD-L1	Stabilize PD-L1 and promote immune escape	+	Evading immune destruction	(Jia et al., 2025)
ZMYND8	Stabilize ZMYND8 and promote the transcription of its target genes ZEB1 and VEGFA	+	Activating invasion and metastasis	(Tang et al., 2024)
SET	Interact with SET and de-stabilize SET	-	Sustaining proliferative signaling	(Chen et al., 2024)
PP2A	Promote the active localization of PP2A in the cytoplasm, inhibit the activity of CDK1	-	Genome instability and mutation	(Galarreta et al., 2021)
Claspin	Remove ubiquitin from Claspin to stabilize it, counteract the degradation by SCF^βTrCP^, and enhance the ATR-Chk1 signaling	+	Resisting cell death	(Faustrup et al., 2009)
TPP1	Stabilize TPP1 and maintain the replication ability of telomeres	+	Enabling replicative immortality	(Zemp and Lingner, 2014)
POT1	Indirectly promote the degradation of POT1 by activating E3 ligases	-	Enabling replicative immortality	(Episkopou et al., 2019)
Yorkie/YAP	Stabilize Yorkie/YAP and inhibit the Hippo tumor-suppressing pathway	+	Evading growth suppressors, sustaining proliferative signaling	(Sun et al., 2019)
HIF-1α	Deubiquitinate HIF-1α and regulate H3K56 acetylation promote hypoxia-induced tumor progression	+	Inducing angiogenesis, activating invasion and metastasis	(Wu et al., 2016)

The oncogenic role of USP7 is currently believed to be primarily mediated through the regulation of the tumor suppressor proteins p53 and PTEN (phosphatase and tensin homolog). Studies have shown that USP7 can deubiquitinate and stabilize the E3 ubiquitin ligase Mdm2, thereby promoting the ubiquitination and subsequent degradation of the substrate p53, which in turn inhibits the tumor suppressive function of p53. Conversely, knockout of USP7 stabilizes p53 (Li et al., 2004). USP7 can also deubiquitinate the tumor suppressor PTEN and alter its cellular localization by promoting its export from the nucleus, thereby inhibiting PTEN function and enhancing PI3K (phosphoinositide 3-kinase) signaling (Song et al., 2008). In prostate cancer patient samples, overexpression of USP7 is negatively correlated with the nuclear export of PTEN. Additionally, research has demonstrated that USP7 deubiquitinates and stabilizes the oncoprotein N-Myc, promoting the progression of neuroblastoma and small cell lung cancer and the inhibition of USP7 suppresses neuroblastoma growth *in vivo* (Tavana et al., 2016; Grunblatt et al., 2020). USP7 also deubiquitinates and stabilizes the oncoprotein KRAS (Kirsten rat sarcoma viral oncogene homolog), thereby promoting the proliferation of non-small cell lung cancer (NSCLC) cells. USP7 inhibitors can suppress the proliferation of NSCLC cells and show particularly pronounced effects in cells resistant to the KRAS-G12C inhibitor AMG510 (Huang et al., 2024). Moreover, USP7 has been reported to participate in the regulation of the Wnt signaling pathway by deubiquitinating and stabilizing β-catenin. USP7 inhibitors can inhibit Wnt signaling and colorectal tumor growth, suppress colorectal cancer cell proliferation, and induce apoptosis (An et al., 2017). Furthermore, USP7 can deubiquitinate and stabilize Yorkie/YAP, thereby inhibiting the Hippo tumor suppressive pathway. In clinical hepatocellular carcinoma (HCC) samples, the expression levels of USP7 and YAP are both upregulated (Sun et al., 2019). USP7 is also involved in regulating angiogenesis, tumor progression, and metastasis. It enhances the stability of HIF-1α through deubiquitination, induces epithelial-mesenchymal transition (EMT), and promotes tumor progression and metastasis via regulation of H3K56 acetylation (Wu et al., 2016).

Under certain conditions, USP7 can exert anti-cancer effects. In contrast to its role in deubiquitinating and stabilizing the E3 ligase Mdm2, USP7 can directly act on p53 to stabilize it. Overexpression of USP7 has been shown to inhibit tumor growth (Li et al., 2002). Thus, the regulatory effect of USP7 on p53 is dual in nature. Studies have reported the use of molecular glues that target both p53 and USP7, linking p53 with the deubiquitinating enzyme USP7, thereby increasing p53 levels and suppressing cancer cell proliferation both *in vitro* and *in vivo* (Li Z. et al., 2024). In this context, the application of molecular glues alters the substrate selectivity of USP7, converting its oncogenic function into an anti-oncogenic one. Similarly, USP7 exerts dual regulatory effects on the Wnt signaling pathway. In addition to its role in deubiquitinating and stabilizing β-catenin, USP7 can also deubiquitinate and stabilize Axin, a scaffold protein of the β-catenin degradation complex, thereby promoting β-catenin degradation and negatively regulating Wnt/β-catenin signaling to inhibit tumor cell proliferation (Ji et al., 2019). Therefore, the impact of USP7 on tumor cells is highly context-dependent, and its function varies depending on the cellular environment or signaling pathway involved.

USP7 plays a key regulatory role in tumor cells, making it an active focus of drug development efforts. Several small-molecule inhibitors targeting USP7 have been reported, including FT671, P5091, OAT-4828, and GNE6776. These inhibitors have demonstrated strong anti-tumor potential in both *in vitro* and preclinical *in vivo* studies. Beyond the traditional inhibitory activity approach, recent research strategies targeting USP7 are shifting toward combination therapy and immune regulation. For example, inhibition of USP7 by P5091 promotes tumor suppression and ferroptosis, enhances the efficacy of the BRAF inhibitor vemurafenib in BRAF^V600E^-mutant thyroid cancer, and helps overcome drug resistance (Hu et al., 2026). FT671 selectively inhibits the activated p53 signaling pathway, and when combined with MEK inhibitors and PD-1 antibodies, it suppresses the initiation and progression of melanoma (Su et al., 2025). FX1-5303, a highly potent and specific USP7 inhibitor, exhibits synergistic effects with the BCL2 inhibitor venetoclax in suppressing the growth of multiple myeloma and acute myeloid leukemia (AML) (Futran et al., 2024). U-20 blocks USP7-mediated stabilization of the immune checkpoint PD-L1 (programmed death ligand 1), reshapes the tumor immune microenvironment, and restores anti-tumor immune function, showing potential for immunotherapy in colorectal cancer (Jia et al., 2025). OAT-4828 is a novel, highly effective oral USP7 inhibitor that alters the tumor microenvironment, enhances anti-tumor immunity, reduces levels of immunosuppressive proteins, for example, PD-L1, and exhibits anti-angiogenic effects. It demonstrates significant anti-cancer activity in models of melanoma, colon cancer, and non-Hodgkin’s lymphoma (Muchowicz et al., 2025; Chrzanowski et al., 2026). USP7 inhibitor GNE6776 reduces EBNA1 (EBV nuclear antigen-1) protein levels in a proteasome-dependent manner, affects chromosome segregation and mitotic cell division pathways in EBV (Epstein-Barr virus) -positive cells, and inhibits the growth of EBV-associated tumors such as gastric cancer and lymphoma (Chen et al., 2025). The successful application of GNE6776 expands the therapeutic potential of USP7 inhibitors into the field of virus-associated tumors, achieving dual anti-viral and anti-tumor effects by interfering with viral protein stability.

In summary, although no USP7-targeting drugs have yet entered clinical trials, several candidate drugs in preclinical studies have demonstrated anti-tumor effects, functioning as both chemotherapy sensitizers and immunomodulators. The core mechanisms include inhibiting USP7 to stabilize p53, regulating MDM2 expression to induce tumor cell apoptosis, and modulating the tumor immune microenvironment. These drugs can also synergistically enhance the efficacy of chemotherapy and radiotherapy, showing promising potential for clinical application. Nevertheless, several research bottlenecks remain for their future clinical development. The main challenges include the following aspects. First, USP7 shares high homology with other deubiquitinases, like USP47, which makes off-target effects likely and may lead to toxicity. Second, inhibition of USP7 may be compensated by other deubiquitinases, potentially reducing therapeutic efficacy. Additionally, pharmacokinetic issues such as the short half-life and low bioavailability of early compounds require further optimization in drug formulation.

## 5 USP22 and cancer

USP22 functions as an integral catalytic component of the SAGA transcriptional co-regulatory complex, wherein it resides specifically within the DUB (Samara et al., 2010). This DUB subcomplex is composed of four constituent polypeptides: USP22, which provides the enzymatic active site, ATXN7, ATXN7L3, and ENY2 (Köhler et al., 2010). Within this assembly, ATXN7L3 acts as a structural adaptor that tethers USP22 to the larger SAGA scaffold, whereas ENY2, a small zinc-finger protein, plays an indispensable role in mediating nucleosomal engagement (Morgan et al., 2016). ATXN7, in turn, serves to bridge the DUB module with the remaining SAGA subunits, thereby positioning USP22 in close spatial proximity to chromatin substrates. Notably, structural investigations have demonstrated that the deubiquitinase activity of USP22 is profoundly dependent on its association with partner proteins within the DUB module; the isolated catalytic domain alone exhibits only marginal enzymatic activity, and its integration into the holocomplex triggers conformational rearrangements that are requisite for full catalytic function (Atanassov et al., 2016). The principal chromatin target of SAGA-associated USP22 is monoubiquitinated histone H2B (H2Bub1), and the removal of this ubiquitin moiety by USP22 constitutes a critical step in promoting both transcriptional initiation and productive elongation (Herbst et al., 2021).

A comprehensive pan-cancer profiling study, examining USP22 expression patterns and genomic aberrations revealed that this protein is widely dysregulated across diverse human neoplastic conditions, with elevated transcript levels being closely associated with advanced clinical stage and diminished overall patient survival (Devi and Shukla, 2026). In parallel, a complementary structural appraisal of USP22 and its interacting regulators has provided further insights into the domain organization, enzymatic mechanism, and protein–protein interaction landscape that collectively underpin its oncogenic potential (Devi and Shukla, 2025). Taken together, these findings firmly position USP22 not only as an essential element of the SAGA transcriptional apparatus but also as a therapeutically relevant proto-oncogenic factor with broad implications across multiple tumor types.

**FIGURE 4:**
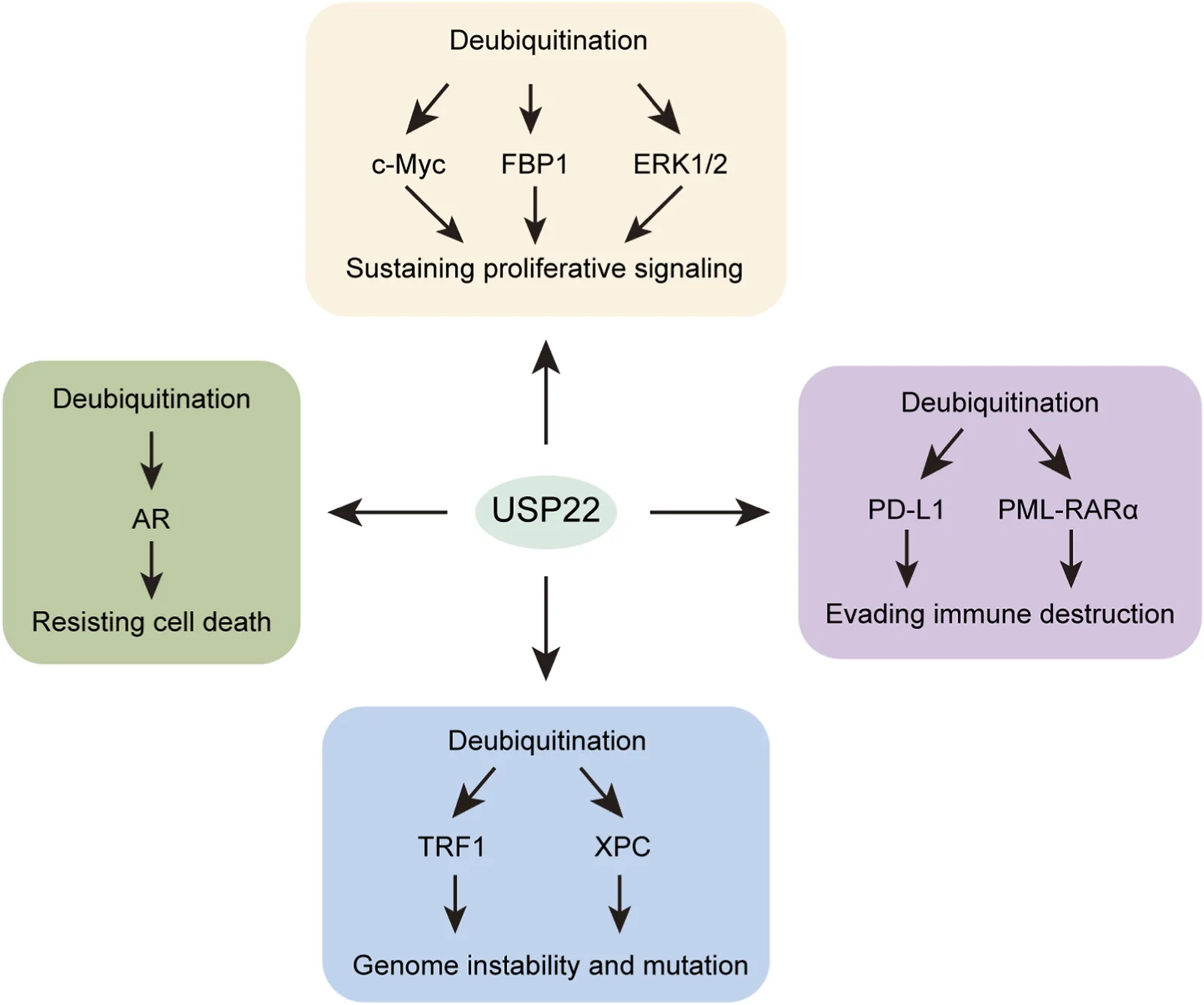
The cancer-related substrates, pathways and cancer hallmarks regulated by USP22. Arrows denote activation or stabilization, bars denote inhibition or degradation.

USP22 -participates in modulating various cancer-related pathways (Figure 4; Table 2) (Zhang et al., 2008; Guo et al., 2022). Research has shown that USP22 is highly expressed in multiple tumor types, and such elevated expression correlates with poor patient prognosis (Glinsky, 2006). In addition, USP22 plays a role in the hallmark of genomic instability and mutation. It deubiquitinates and stabilizes TRF1, a core component of the telomere protection protein complex. This regulatory function requires its association with the SAGA complex (Atanassov et al., 2009).

**TABLE 2:** Substrates, pathways and cancer hallmarks regulated by USP22.

Regulated substrates	Function of mechanism	Tumor promotion (+)/tumor suppression (−)	Cancer hallmarks involved	References
TRF1	Stabilize TRF1	+	Genome instability and mutation	(Atanassov et al., 2009)
c-Myc	Stabilize c-Myc and inhibit the proteolysis of c-Myc	+	Sustaining proliferative signaling	(Kim et al., 2017)
AR	Regulate the accumulation of AR-related proteins and signaling pathways, and further enhance the expression of target genes regulated by both AR and MYC	+	Resisting cell death, sustaining proliferative signaling	(Schrecengost et al., 2014)
Unclear	Reduce the activity of mTOR	-	Sustaining proliferative signaling	(Kosinsky et al., 2020)
PD-L1	Directly regulate the stability of PD-L1 and control the protein level of PD-L1 through the USP22-CSN5-PD-L1 axis	+	Evading immune destruction	(Wang et al., 2020a)
XPC	Stabilize XPC, regulate cell survival and DNA repair	+	Genome instability and mutation, sustaining proliferative signaling	(McCann et al., 2020)
FBP1	Deubiquitinate transcriptional regulatory factor FBP1 and regulate cell proliferation	+	Sustaining proliferative signaling	(Atanassov and Dent, 2011)
PML-RARα	Deubiquitinate and stabilize PML-RARα	+	Evading immune destruction, resisting cell death	(Kowald et al., 2024)
ERK1/2	Deubiquitinate and stabilize ERK1/2	+	Sustaining proliferative signaling	(Ma et al., 2026)

In addition, USP22 has also been reported to regulate multiple cancer-related targets in the cytoplasm, potentially through mechanisms independent of the SAGA complex. USP22 positively regulates the stability and tumorigenic activity of c-Myc in both mammalian systems and breast cancer cells. In various breast cancer cell lines, USP22 promotes the deubiquitination of c-Myc, thereby increasing its protein levels (Kim et al., 2017). As a deubiquitinating enzyme, USP22 directly stabilizes the androgen receptor (AR), enhancing its protein accumulation and signaling pathway activity, and further upregulates the expression of target genes co-regulated by AR and MYC. Collectively, these functions establish USP22 as a key effector factor in tumor progression, driving a lethal phenotype (Schrecengost et al., 2014).

USP22 can also regulate PD-L1 levels in cells. It interacts with PD-L1 and enhances its stability by inhibiting proteasome-mediated degradation. Additionally, USP22 interacts with CSN5 and stabilizes it through deubiquitination, thereby indirectly regulating PD-L1 protein levels. Loss of USP22 suppresses tumor development and promotes T cell cytotoxicity. In human non-small cell lung cancer samples, USP22 expression is positively correlated with PD-L1 expression (Wang et al., 2020a). USP22 also plays a role in genotoxic damage. Its absence renders cells more sensitive to such damage. By regulating XPC, USP22 influences cell survival and DNA repair, thereby exerting a carcinogenic driving effect in prostate cancer (McCann et al., 2020). Furthermore, USP22 regulates the ubiquitination of far upstream element (FUSE)-binding protein 1 (FBP1), stabilizing its stable recruitment at target gene sites and thereby controlling cell proliferation (Atanassov and Dent, 2011). In acute promyelocytic leukemia (APL), USP22 deubiquitinates and stabilizes the APL fusion protein PML-RARα, inhibits interferon (IFN) effects, and promotes differentiation, contributing to carcinogenesis (Kowald et al., 2024). In contrast to its oncogenic activities, USP22 has also been reported to reduce mTOR activity and exert a tumor-suppressive role in colorectal cancer. Loss of USP22 leads to increased mTOR activity, which exacerbates the tumor burden in this cancer type (Kosinsky et al., 2020).

In summary, USP22, a DUB encoded by a ‘cancer death gene’, primarily exerts its oncogenic functions during cancer development and progression. By regulating key cancer-related pathways and proteins such as c-Myc, PD-L1, TRF1, and FBP1, USP22 is involved in the modulation of multiple cancer hallmarks, including sustaining proliferative signaling, avoiding immune destruction, and inducing genome instability and mutation. Consequently, USP22 promotes the initiation and progression of various cancers, such as breast cancer, lung cancer, prostate cancer, and APL. Furthermore, studies have revealed that USP22 exhibits tumor- and environment-dependent specific effects, indicating that its expression level may serve as a biomarker in clinical treatment (Kosinsky et al., 2020).

In recent years, significant progress has also been made in the development of selective inhibitors targeting USP22. To date, several small-molecule compounds with well-defined inhibitory activity have been reported, some of which have advanced to the preclinical research stage. The development of USP22 inhibitors spans multiple cancer types, with particular emphasis on their potential in tumor immunotherapy (Zhou. et al., 2026). Zhang et al. identified Rottlerin (IC_50_ = 2.53 μM) and Morusin (IC_50_ = 8.29 μM) as selective and potent USP22 inhibitors. Treatment with Rottlerin or Morusin increases the ubiquitination level of H2B while reducing the expression of Sirt1 and PD-L1 in a USP22-dependent manner. In syngeneic mouse tumor models, both Rottlerin and Morusin exhibited strong antitumor activity, accompanied by enhanced infiltration of T cells into tumor tissues, positioning them as potential drug candidates for cancers such as colorectal cancer and melanoma (Zhang et al., 2023). Ma et al. reported that ACT001, a small-molecule compound, targets USP22. It inhibits USP22-induced ubiquitination of ERK1/2, leading to subsequent ERK1/2 degradation, thereby effectively suppressing the growth of colorectal cancer cells both *in vitro* and *in vivo* (Ma et al., 2026).

In addition to the synthetic small molecule compounds obtained through screening, the natural compound gentiopicroside has also been reported to be a potent USP22 inhibitor with anti-tumor immune therapeutic activity. Molecular docking and molecular dynamics simulations have demonstrated that gentiopicroside can stably bind to the catalytic pocket of USP22, confirming it as a USP22 inhibitor. Administration of gentiopicroside to mice significantly enhances anti-tumor immunity and thereby inhibits the growth of orthotopically transplanted lung adenocarcinoma (Lu W. et al., 2024). Furthermore, the marketed drug ergotamine exhibits high binding affinity and specific interactions with the USP22 binding pocket. Through all-atom molecular dynamics simulations, the molecular mechanism underlying its inhibitory activity against USP22 has been elucidated. As a new application of an old drug, ergotamine holds potential for future cancer treatment, although further experimental validation is still required (Alshehri et al., 2025).

Overall, significant progress has been made in the development of inhibitors targeting USP22, with several promising compounds currently in active preclinical research stages. Among them, candidate molecules such as gentiopicroside and ACT001 have demonstrated potent anti-tumor immune activity in animal models, laying a solid foundation for future clinical translation.

## 6 USP10 and cancer

**FIGURE 5:**
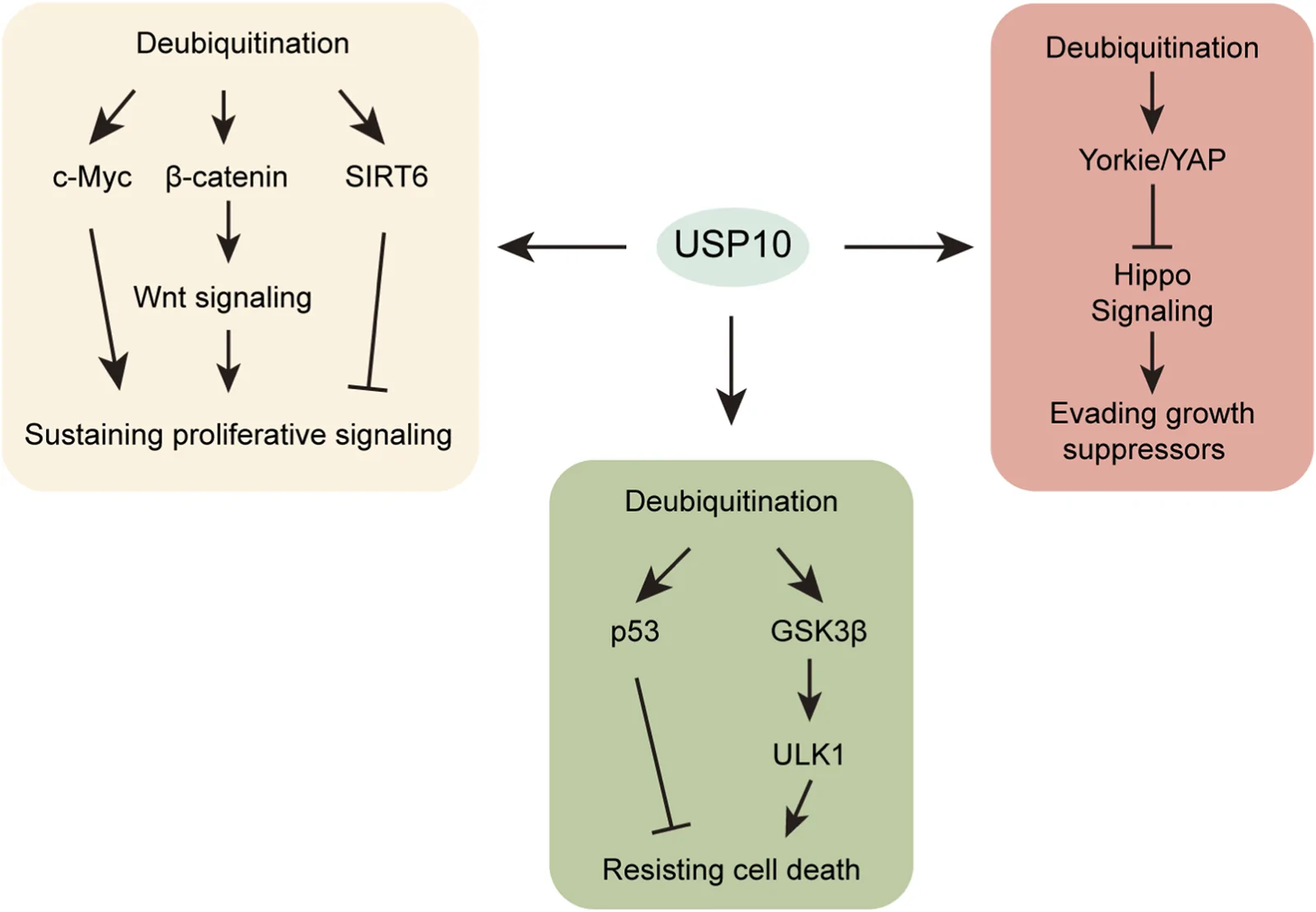
The cancer-related substrates, pathways and cancer hallmarks regulated by USP10. Arrows denote activation or stabilization, bars denote inhibition or degradation.

USP10 is a highly abundant and widely expressed DUB in cells. It has been reported to be highly expressed in various tumor tissues and associated with poor patient prognosis, suggesting a potential tumor-promoting function. USP10 is involved in regulating key oncogenes and tumor suppressor genes, such as p53 and Myc (Figure 5; Table 3). Specifically, USP10 can deubiquitinate the tumor suppressor p53, reversing the nuclear export and degradation of p53 induced by Mdm2, thereby altering the localization and stability of p53. Following DNA damage, the stability of USP10 increases, and a portion of USP10 translocates to the nucleus to activate p53. Thus, USP10 plays a tumor-suppressive role in this context, and its expression is significantly downregulated in renal clear cell carcinoma cells (Yuan et al., 2010). Additionally, USP10 participates in regulating Wnt signaling pathway transduction. It can deubiquitinate and stabilize β-catenin, thereby maintaining undifferentiated tumor characteristics. This function contributes to the properties of colorectal cancer cells, stemness maintenance, and tumorigenic growth, and leads to aberrant Wnt signaling, thereby promoting tumor initiation and progression (Reissland et al., 2024).

**TABLE 3:** Substrates, pathways and cancer hallmarks regulated by USP10.

Regulated substrates	Function of mechanism	Tumor promotion (+)/tumor suppression (−)	Cancer hallmarks involved	References
p53	Reverse the process of Mdm2-induced nuclear export and degradation of p53	-	Resisting cell death	(Yuan et al., 2010)
β-catenin	Deubiquitinate and stabilizeβ-catenin	+	Sustaining proliferative signaling	(Reissland et al., 2024)
c-Myc	Stabilize c-Myc and inhibit the proteolysis of c-Myc	+	Sustaining proliferative signaling	(Li et al., 2026a)
SIRT6	Deubiquitinate and stabilize the Myc transcriptional inhibitory factor SIRT6	-	Sustaining proliferative signaling	(Lin et al., 2013)
GSK3β	Stabilize GSK3β and promote the transcription of ULK1	+	Resisting cell death, activating invasion and metastasis	(Feng et al., 2024)
Yorkie	Stabilize Yorkie and inhibit the Hippo tumor suppressor pathway	+	Evading growth suppressors, sustaining proliferative signaling, resisting cell death	(Gao et al., 2019)
YAP	Stabilize YAP and inhibit the Hippo tumor suppressor pathway	+	Sustaining proliferative signaling, evading growth suppressors	(Zhu et al., 2020)
YAP	Stabilize YAP, inhibit its degradation, and upregulate p53 and p21	+	Sustaining proliferative signaling, resisting cell death	(Lu et al., 2024b)

USP10 exerts a bidirectional regulatory effect on the Myc protein. On one hand, USP10 can directly remove ubiquitin chains from the c-Myc protein, thereby preventing its proteasome-dependent degradation. This leads to the stabilization and upregulation of c-Myc protein levels, contributing to a carcinogenic effect (Li A. et al., 2026). On the other hand, USP10 also plays a tumor-suppressive role by modulating the transcriptional activity of the Myc protein. Specifically, USP10 deubiquitinates and stabilizes SIRT6 (sirtuin 6), a known transcriptional repressor of Myc, thereby inhibiting cell cycle progression, cancer cell growth, and tumor formation. Notably, in colon cancer tissues, the protein expression levels of both USP10 and SIRT6 are significantly reduced (Lin et al., 2013).

In addition, USP10 is also involved in regulating the Hippo-YAP signaling pathway. It can deubiquitinate and stabilize the transcriptional co-activators Yorkie and YAP, thereby promoting cell proliferation and inhibiting apoptosis. In liver cancer tissues, the expression level of USP10 is positively correlated with that of YAP, and the tumor-promoting role of USP10 in liver cancer development has been validated in animal models (Gao et al., 2019; Zhu et al., 2020). Furthermore, research on osteosarcoma has revealed that USP10 stabilizes GSK3β (glycogen synthase kinase 3β), which in turn promotes ULK1 transcription, induces autophagy, and enhances cell proliferation and invasion. The ability of USP10 to accelerate osteosarcoma progression *in vivo* has also been confirmed (Feng et al., 2024). Therefore, USP10 plays a ‘double-edged sword’ role in human cancers. However, owing to the complexity and diversity of cellular environments, the precise molecular mechanisms underlying its context-dependent effects on tumorigenesis remain unclear. Notably, the oncogenic effect of USP10, mediated through the regulation of key proteins, positions it as a potential target for cancer therapy (Tao et al., 2022). Nevertheless, attention should also be paid to its tumor-suppressive function under specific background conditions.

In recent years, several selective inhibitors targeting USP10 have been developed. Yu et al. reported the USP10-targeting inhibitor Wu-5, which inhibits the FLT3 and AMPK signaling pathways, overcomes resistance to FLT3 inhibitors, and enhances crenolanib-induced death of FLT3-ITD-positive acute myeloid leukemia cells (Yu et al., 2021). Lu et al. reported the small-molecule compound D1, which targets USP10 and promotes YAP degradation, thereby downregulating the expression of p53 and its downstream protein p21. This leads to S-phase cell cycle arrest and induces apoptosis in HCC cells. Studies have shown that D1 significantly inhibits the proliferation and colony formation of HCC cells (Lu Y. et al., 2024). Based on D1, the compound LY-2 was further developed. Through systematic structure-activity relationship analysis, LY-2 was found to exhibit significantly enhanced binding affinity compared to D1, making it the first USP10 inhibitor with nanomolar binding affinity. LY-2 effectively inhibits USP10-mediated proteasomal degradation of downstream proteins YAP and p53, thereby downregulating CDK4 in the p53 signaling pathway, promoting apoptosis of HCC cells, and suppressing the initiation and progression of liver cancer (Lu et al., 2025). Subsequently, a novel water-soluble USP10 inhibitor, WW-104, with improved solubility, was further optimized based on LY-2. WW-104 significantly inhibits the proliferation and colony formation of HCC cells and induces G2 phase arrest and apoptosis (Wang et al., 2026). Collectively, the above series of studies have revealed the potential of USP10 as a therapeutic target for liver cancer and other diseases. The successful development of selective inhibitors targeting USP10, particularly compounds D1, LY-2, and WW-104, represents milestone progress in the research and development of selective USP10 inhibitors, positioning them as candidate molecules with therapeutic potential. Furthermore, as tool compounds, they will facilitate further research on the biological functions of USP10 in various diseases.

## 7 USP35 and cancer

**FIGURE 6:**
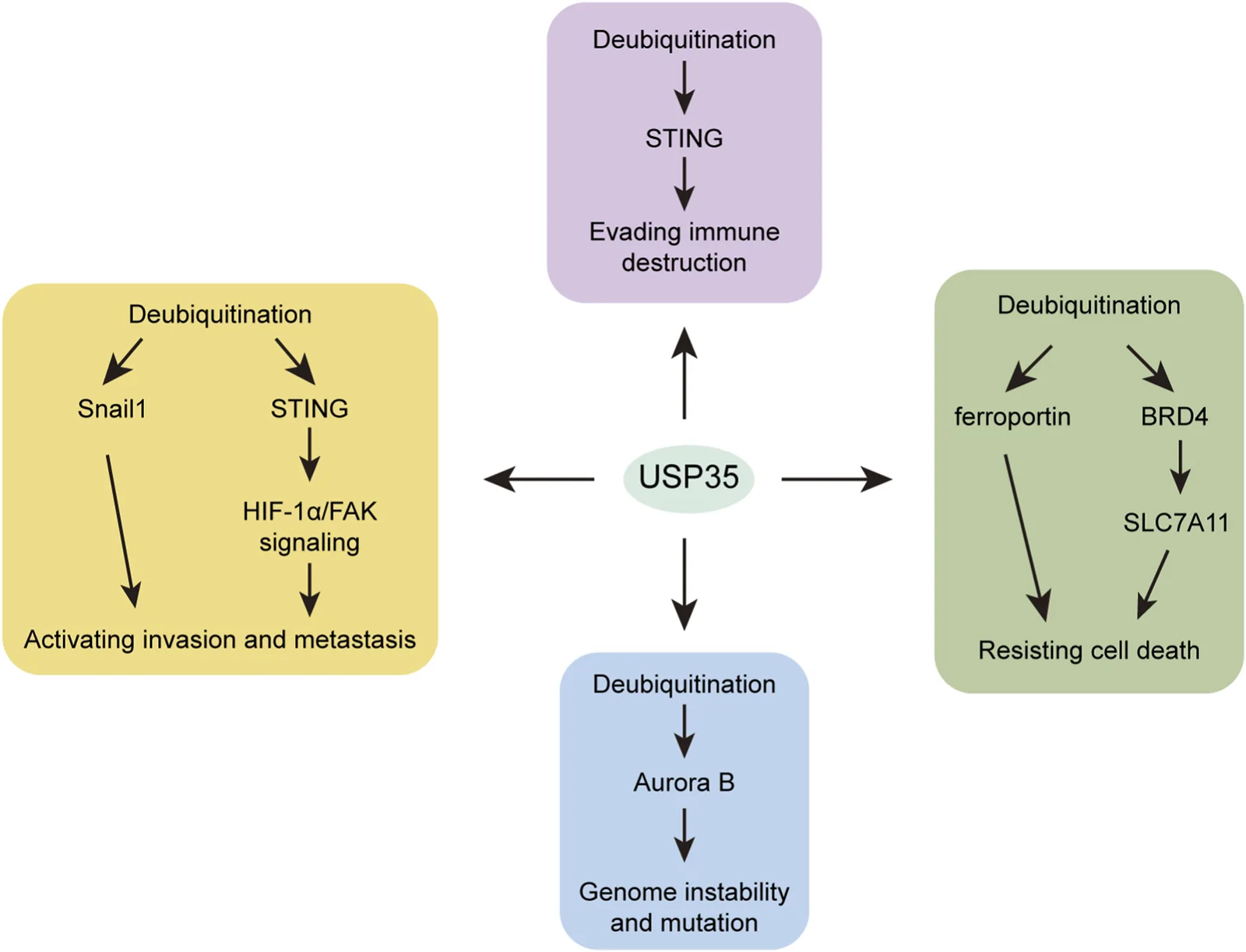
The cancer-related substrates, pathways and cancer hallmarks regulated by USP35. Arrows denote activation or stabilization, bars denote inhibition or degradation.

USP35 is a DUB of particular interest in the context of cancer occurrence and progression. Bioinformatics analyses have revealed that USP35 is significantly overexpressed in nine types of cancer, including gastric cancer and thyroid cancer. Furthermore, its expression level is closely associated with tumor stage, metastasis, and poor prognosis (Yan et al., 2025b; Lian et al., 2025; Xiao et al., 2023; Guo et al., 2025; Liu C. et al., 2022), suggesting that USP35 may function as a cancer-promoting protein and play a role in the development and progression of various cancers (Figure 6; Table 4).

**TABLE 4:** Substrates, pathways and cancer hallmarks regulated by USP35.

Regulated substrates	Function of mechanism	Tumor promotion (+)/tumor suppression (−)	Cancer hallmarks involved	References
Aurora B	Stabilize Aurora B and regulate the mitotic process	+	Genome instability and mutation	(Park et al., 2018)
Ferroportin	Stabilize ferroportin and regulate ferroptosis	+	Resisting cell death	(Tang et al., 2021)
Snail1	Stabilize Snail1 and promote tumor metastasis	+	Activating invasion and metastasis	(Ma et al., 2024)
BRD4	Stabilize BRD4 and upregulate the expression of SLC7A11	+	Resisting cell death	(Cao et al., 2025)
STING	Deubiquitinate and inactivate STING	+	Evading immune destruction	(Zhang et al., 2021)
STING	Deubiquitinate and stabilize STING, and activate the HIF-1α/FAK pathway	+	Activating invasion and metastasis, reprogramming energy metabolism	(Yan et al., 2025a)

USP35 plays a regulatory role in cell mitosis. It deubiquitinates and stabilizes Aurora B, thereby protecting it from APCCDH1-mediated proteasomal degradation and maintaining its steady-state levels during mitosis (Park et al., 2018). However, the impact of USP35 on cell cycle regulation and its associated carcinogenic effects require further validation. Additionally, USP35 is involved in the regulation of ferroptosis. It is highly expressed in human lung cancer tissues and cell lines, where it deubiquitinates and stabilizes the iron transporter ferroportin (FPN). Knockdown of USP35 promotes ferroptosis and inhibits the cell growth, colony formation, and tumor progression of lung cancer cells, while also enhancing their sensitivity to cisplatin and paclitaxel chemotherapy (Tang et al., 2021). Moreover, USP35 suppresses ferroptosis in ER-positive breast cancer cells. Specifically, USP35 interacts with BRD4 and stabilizes the BRD4 protein through deubiquitination, which in turn upregulates SLC7A11 expression, thereby inhibiting ferroptosis and promoting the growth of ER-positive breast cancer cells (Cao et al., 2025).

USP35 has also been reported to be associated with the progression of gastric cancer. It promotes metastasis and disease progression by deubiquitinating and stabilizing Snail1, a key transcription factor involved in EMT, thereby positioning USP35 as a potential therapeutic target for gastric cancer (Ma et al., 2024). Furthermore, in gastric cancer, USP35 deubiquitinates and stabilizes STING, leading to activation of the HIF-1α/FAK pathway and promotion of energy metabolism reprogramming. These events enhance the adhesive capacity of gastric cancer cells and facilitate peritoneal dissemination (Yan et al., 2025a). In ovarian cancer research, USP35 was found to directly deubiquitinate STING and subsequently inactivate it. Silencing USP35 enhances the activation of the STING-TBK1-IRF3 pathway and promotes the expression of type I interferons. Furthermore, knockdown of USP35 increases the sensitivity of ovarian cancer cells to the DNA-damaging chemotherapeutic agent cisplatin (Zhang et al., 2021). Interestingly, when comparing the findings in gastric cancer and ovarian cancer, USP35 has been reported to deubiquitinate STING but exert opposing effects, either stabilization or inactivation, depending on the tumor context. Despite these differences, USP35 consistently plays a tumor-promoting role across both cancer types.

Furthermore, studies have shown that USP35 has two distinct isoforms, which localize to different subcellular compartments and exhibit different functions. Isoform 1 inhibits apoptosis induced by staurosporine and TNF-related apoptosis-inducing ligand (TRAIL, also known as TNFSF10), whereas isoform 2 mediates cell death triggered by endoplasmic reticulum stress (Leznicki et al., 2018). The regulatory roles of the different USP35 isoforms in tumors still require further investigation. As USP35 has garnered attention as a potential anti-cancer target in recent years (Li B. et al., 2026), the development of USP35-targeted inhibitors remains at an early stage, and no highly selective inhibitors targeting USP35 have yet been identified through high-throughput screening. Given that USP35 is highly expressed in multiple cancer types and participates in the regulation of various tumor characteristics and processes, an efficient and selective inhibitor of USP35 holds promise as a future target for tumor detection and therapy.

## 8 USP4 and cancer

**FIGURE 7:**
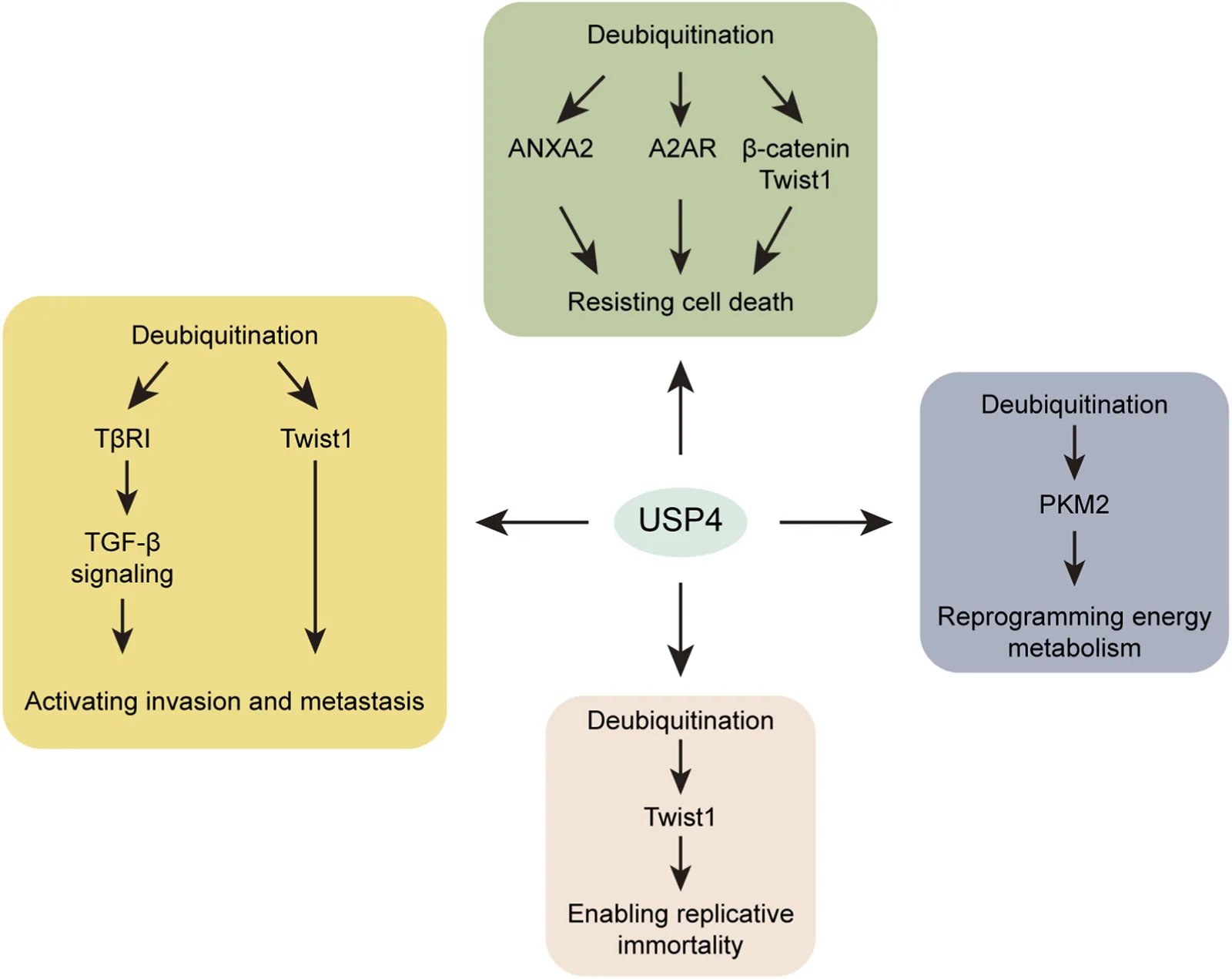
The cancer-related substrates, pathways and cancer hallmarks regulated by USP4. Arrows denote activation or stabilization, bars denote inhibition or degradation.

USP4 regulates a variety of classic cancer-related signaling pathways, thereby playing an important role in several pathological and physiological processes, including tumor initiation and progression. In recent years, studies have revealed that USP4 exerts multiple regulatory effects on the proliferation, migration, invasion, and apoptosis of diverse tumor cell types (Figure 7; Table 5). Furthermore, USP4 can serve as a prognostic biomarker for various cancers (Wang et al., 2020b). For instance, research related to breast cancer has demonstrated that the PAK5-DNPEP-USP4 signaling axis regulates breast cancer growth and metastasis. Analysis of clinical breast cancer samples showed markedly elevated levels of PAK5 and USP4, and the high expression of these two proteins was associated with a poorer survival rate in breast cancer patients (Geng et al., 2020).

**TABLE 5:** Substrates, pathways and cancer hallmarks regulated by USP4.

Regulated substrates	Function of mechanism	Tumor promotion (+)/tumor suppression (−)	Cancer hallmarks involved	References
TβRI	Stabilize TβRI and activate the TGF-β signaling pathway	+	Activating invasion and metastasis	(Zhang et al., 2012)
ANXA2	Stabilize and activate ANXA2	+	Resisting cell death	(Tu et al., 2025)
A2AR	Stabilize A2AR, regulate autophagy and inhibit ferroptosis	+	Resisting cell death	(Zhao et al., 2025)
β-catenin, Twist1	Stabilize β-catenin and Twist1, inhibit their degradation, and enhance the characteristics of tumor cell stem cells	+	Resisting cell death	(Li et al., 2025)
PKM2	Stabilize PKM2 and promote the proliferation of gastric cancer cells and glucose metabolism	+	Reprogramming energy metabolism	(Chen et al., 2023b)
Twist1	Deubiquitinate and stabilize Twist1	+	Activating invasion and metastasis	(Xu et al., 2023)
Twist1	Deubiquitinate and stabilize Twist1	+	Enabling replicative immortality	(Li et al., 2020a)

From the perspective of regulatory mechanisms, USP4 has been identified as a potent activator of the TGF-β signaling pathway. It directly interacts with TβRI (transforming growth factor-β type I receptor) and functions as a deubiquitinating enzyme, thereby regulating TβRI levels on the cell membrane. Knockdown of USP4 inhibits TGF-β-induced EMT and tumor metastasis. USP4 is also characterized by upregulated expression in breast cancer cells and tissues (Zhang et al., 2012). Further studies have shown that lactate promotes the deubiquitination of USP4 and stabilizes ANXA2, which facilitates glioblastoma progression and confers stem cell-like properties to glioblastoma stem cells (GSCs), thereby promoting GSC maintenance and radioresistance (Tu et al., 2025). In gastric cancer, USP4 deubiquitinates and stabilizes A2AR, suppresses autophagy, and subsequently inhibits ferroptosis, thus promoting tumor progression. Targeting USP4 or A2AR activates autophagy and restores ferroptosis, indicating that the USP4-A2AR signaling axis is critical for gastric cancer cell survival (Zhao et al., 2025). Additionally, USP4 interacts with PKM2 (pyruvate kinase M2) and catalyzes its deubiquitination, thereby promoting proliferation, glucose uptake, and lactate production in gastric cancer cells. Knockdown of USP4 reduces PKM2 levels, leading to decreased cell proliferation and glycolytic activity (Chen Y. et al., 2023).

Moreover, the regulatory role of USP4 on Twist1 has been reported in multiple studies. The FBXO3-USP4-Twist1 signaling axis plays a key role in PI3K-mediated breast cancer metastasis. FBXO3 binds to and stabilizes USP4, which in turn enhances Twist1 protein stability, thereby promoting the migration and metastasis of breast cancer cells (Xu et al., 2023). USP4 is also critical for promoting stem cell characteristics in lung cancer. It binds to Twist1 and stabilizes the Twist1 protein through deubiquitination. USP4 is highly expressed in human lung cancer specimens and shows a positive correlation with Twist1 expression. High expression of USP4/Twist1 is associated with a poorer clinical prognosis in lung cancer patients, suggesting that USP4 may serve as a potential target for the diagnosis and treatment of lung cancer (Li F. et al., 2020).

Therefore, in light of the extensive pro-cancer effects reported for USP4, this deubiquitinating enzyme has emerged as a promising candidate for targeted cancer therapy. In 2025, Li et al. reported the discovery of U4-I05, a selective natural small-molecule inhibitor of USP4 (Li et al., 2025). By binding to the C311 active site of USP4, U4-I05 induces the degradation of β-catenin and Twist1 proteins, thereby suppressing cancer stem cell traits and chemoresistance. This finding provides a direct pharmacological tool for targeting USP4. In a genetically engineered mouse model of metastatic colorectal cancer (CRC), treatment with U4-I05 effectively inhibited tumor metastasis and extended survival, demonstrating significant therapeutic efficacy against CRC. This study opens up promising avenues for targeting USP4 in cancer therapy and for developing novel therapeutic strategies aimed at cancer stem cell characteristics and chemoresistance. Further development of selective USP4 inhibitors is warranted to provide both pharmacological research tools and a foundation for clinical cancer treatment.

## 9 DUBs in tumor microenvironment remodeling

Beyond their intrinsic oncogenic functions within tumor cells, DUBs actively participate in the remodeling of the tumor microenvironment (TME) by modulating immune cell infiltration, cytokine secretion profiles, and the expression of immune checkpoint molecules. The regulatory impact of DUBs on the TME is particularly well-documented in highly immunosuppressive malignancies, such as pancreatic ductal adenocarcinoma (PDAC), where they influence not only malignant progression but also the immune landscape (Zhou et al., 2025). To date, numerous members of the USP family and other DUBs have been identified as critical regulators of the TME, collectively facilitating immune evasion and tumor malignant progression.

Among the TME-regulatory network, USP7 has garnered considerable attention. In tumor-associated macrophages (TAMs), USP7 promotes the phenotypic reprogramming of macrophages from the M2 (immunosuppressive) toward the M1 (pro-inflammatory) subtype through activation of the p38 MAPK signaling cascade, thereby potentiating antitumor immune responses (Hsu et al., 2024). In microsatellite-stable (MSS) colorectal cancer, pharmacological inhibition of USP7 markedly upregulates the expression of chemokines CXCL9, CXCL10, and CXCL11, which in turn facilitate the recruitment of CD8^+^ T lymphocytes into the tumor bed, converting immunologically “cold” tumors into “hot” ones and consequently improving the responsiveness to immune checkpoint blockade therapies (Gao et al., 2023). Collectively, these findings underscore the therapeutic potential of USP7 inhibitors as immunomodulatory agents.

USP22, in contrast, contributes to the establishment of an immunosuppressive TME through a dual-pathway mechanism. On one hand, USP22 stabilizes PD-L1 on the tumor cell surface via the USP22-CSN5-PD-L1 axis, directly dampening T cell-mediated cytotoxicity (Dai et al., 2020). More specifically, USP22 directly interacts with the C-terminus of PD-L1, inducing its deubiquitination and stabilization, and its genetic depletion has been shown to increase tumor immunogenicity and tumor-infiltrating lymphocytes in liver cancer models (Huang et al., 2019). On the other hand, it stabilizes the transcription factor FOXP3 within regulatory T cells (Tregs), reinforcing their suppressive functionality and further exacerbating immune evasion (Yin et al., 2025). Given its concurrent targeting of both tumor-intrinsic PD-L1 stabilization and Treg-mediated inhibitory circuits, USP22 represents a pivotal nodal point in the immunomodulatory network of the TME.

In addition to USP22, several other DUBs have been identified as key regulators of PD-L1 stability and immune suppression. For instance, USP2 has been identified as a novel regulator of PD-L1 stabilization in colorectal and prostate cancer cells. USP2 directly interacts with PD-L1 and deconjugates K48-linked polyubiquitination at lysine 270, thereby preventing its endoplasmic reticulum-associated degradation. Consequently, USP2 ablation enhances antitumor immunity by increasing CD8^+^ T cell infiltration and reducing immunosuppressive MDSCs and Tregs, highlighting USP2 as a potential therapeutic target for cancer immunotherapy (Kuang et al., 2023). Similarly, USP27X has been identified as a critical regulator of PD-L1 stability in lung cancer, where it interacts with and stabilizes PD-L1. Loss of USP27X leads to rapid PD-L1 degradation, increased immune cell infiltration, and enhanced T-cell infiltration, suggesting that targeting USP27X could destabilize PD-L1 and improve immunotherapy efficacy (Singh et al., 2026).

Furthermore, USP8 has been reported to exert a more complex, dual-faceted role in TME remodeling. While originally cited for activating the TRAF6/NF-κB axis to promote pro-inflammatory cytokine production (Guo et al., 2022), recent evidence demonstrates that USP8 inhibition actually enhances anti-PD-1/PD-L1 immunotherapy efficacy by reshaping an inflamed TME. Mechanistically, USP8 inhibition increases PD-L1 protein abundance via TRAF6-mediated K63-linked ubiquitination, which antagonizes its K48-linked degradation, while also triggering innate immune responses and MHC-I expression through NF-κB activation. Thus, combining USP8 inhibitors with PD-1/PD-L1 blockade synergistically activates CD8^+^ T cells to suppress tumor growth (Xiong et al., 2022).

In addition to the above-mentioned members, a broader spectrum of DUBs has also been implicated in TME remodeling. USP1 has been reported to facilitate the activation of TAMs and drive their polarization toward a tumor-supportive phenotype (Li J. et al., 2020). Furthermore, the deubiquitinase ATXN3 stabilizes the transcription factor JunB, which subsequently induces PD-L1 expression, contributing to the upregulation of immune checkpoints within the TME (Montauti et al., 2022). In the context of PDAC, a growing body of evidence indicates that DUBs modulate not only tumor cell proliferation, migration, and chemoresistance but also the immune microenvironment, underscoring their broad regulatory reach across different tumor types (Zhou et al., 2026). These examples collectively illustrate that the functional repertoire of DUBs in TME regulation extends well beyond a limited set of enzymes, encompassing a considerably broader and intricately interconnected deubiquitinase network, the full scope and depth of which warrant further systematic investigation.

## 10 DUB-mediated therapeutic resistance: mechanistic diversity and combinatorial intervention strategies

DUBs contribute to the emergence and maintenance of therapeutic resistance in tumors through a variety of functionally distinct molecular pathways, extending their regulatory scope to the homeostatic control of immune checkpoint molecules, evasion from kinase inhibitor efficacy, active avoidance of ferroptosis, and acquired resistance driven by EMT and stemness-associated traits. A systematic dissection of the molecular underpinnings of these resistance pathways not only deepens our understanding of the adaptive survival mechanisms of tumors, but also furnishes a theoretical foundation and strategic guidance for the rational integration of DUB inhibitors into existing antineoplastic regimens.

### 10.1 DUB-dependent stabilization of PD-L1 and immunotherapy resistance

Aberrant overexpression of the immune checkpoint molecule PD-L1 on the tumor cell surface represents a core event mediating immune evasion and resistance to immune checkpoint inhibitors. In recent years, accumulating evidence has demonstrated that multiple DUBs can effectively elevate the membrane expression level of PD-L1 by removing ubiquitin chains from the PD-L1 protein and suppressing its proteasome-dependent degradation, thereby assisting tumor cells in escaping immune surveillance. Specifically, USP5, USP22, USP14, and the non-USP family member CSN5 have all been validated as possessing the capacity to deubiquitinate and stabilize PD-L1 (Xie et al., 2025). Loss-of-function experiments have further revealed that pharmacological inhibition or genetic ablation of the aforementioned DUBs significantly reduces PD-L1 protein abundance, restores the cytolytic activity of T cells, and synergistically potentiates the antitumor efficacy of anti-PD-1/PD-L1 therapies (Zhang et al., 2024). Collectively, these findings suggest that targeting DUB-mediated PD-L1 stabilization may serve as an effective ancillary strategy for sensitizing tumors to immunotherapeutic intervention.

### 10.2 Contribution of DUBs to kinase inhibitor resistance

In the realm of targeted therapy, DUBs have likewise exhibited non-negligible roles in driving drug resistance. USP7 maintains the protein stability of FK506-binding protein 4 (FKBP4) through its deubiquitinating activity, and this pathway has been closely linked to acquired resistance to the third-generation EGFR-TKI osimertinib in patients with EGFR-mutant non-small cell lung cancer. Functional intervention studies have shown that inhibition of USP7 activity effectively reduces FKBP4 levels and restores the sensitivity of resistant cells to osimertinib (Pang et al., 2026). In a parallel manner, USP47 drives the maintenance of a resistant phenotype by stabilizing the transcription factor YB-1, a critical effector molecule in imatinib resistance in chronic myeloid leukemia (CML), whereas blockade of USP47 attenuates YB-1 protein expression and resensitizes CML cells to imatinib treatment (Lei et al., 2021). These studies collectively delineate a general paradigm in which DUBs mediate acquired resistance across distinct tumor types and targeted therapeutic contexts through the regulation of specific substrate stability.

### 10.3 DUB regulatory networks in ferroptosis evasion and chemoresistance

Ferroptosis, an iron-dependent form of regulated cell death, has emerged as a critical effector pathway underlying the efficacy of various chemotherapeutic agents and targeted compounds. The capacity of tumor cells to resist ferroptosis is closely and functionally linked to their tolerance to conventional therapies, with DUBs playing pivotal roles in this resistance process. USP8 protects tumor cells from ferroptotic insults by deubiquitinating and stabilizing GPX4, the most central negative regulatory enzyme in the ferroptosis pathway (Li H. et al., 2024). USP35 suppresses ferroptosis through two independent yet complementary mechanisms. First, by stabilizing ferroportin to reduce the intracellular labile iron pool. Second, by upregulating SLC7A11 expression via the BRD4 signaling axis to promote glutathione synthesis and storage (Tang et al., 2021). Furthermore, USP11 reinforces the ferroptosis-tolerant phenotype of tumor cells by stabilizing the transcription factor NRF2, a master regulator of the antioxidant response that drives the transcriptional activation of multiple anti-ferroptotic genes (Cao et al., 2025). Collectively, these observations indicate that DUBs constitute a multi-tiered protective barrier within the ferroptosis regulatory network, the functional redundancy and synergy of which warrant further in-depth exploration.

### 10.4 DUB-dependent resistance mechanisms associated with EMT and stemness characteristics

The EMT process and the acquisition of cancer stem cell (CSC) properties are widely recognized as critical cellular bases for intrinsic and acquired chemoresistance in tumors, and the regulatory involvement of DUBs in these processes has attracted increasing attention. USP4 maintains Twist1 protein stability through the FBXO3-USP4-Twist1 signaling axis, thereby driving the initiation of the EMT program and reinforcing cancer stem cell-like traits, ultimately conferring marked chemoresistance upon tumor cells (Li F. et al., 2020). Concurrently, USP35 stabilizes Snail1, another key EMT-associated transcription factor, not only enhancing the invasive and migratory capacity of tumors but also synergistically promoting cross-resistance to multiple chemotherapeutic agents (Li et al., 2025). These lines of evidence suggest that DUBs, by targeting distinct EMT transcription factors, constitute a functionally overlapping yet differentially weighted regulatory network governing drug resistance.

In summary, DUBs participate in the holistic regulation of tumor resistance through a networked mode of action, operating across multiple dimensions including immune evasion, targeted drug sensitivity, oxidative death tolerance, and cellular plasticity. A central translational implication of these mechanisms is that pharmacological inhibition of specific DUBs may resensitize refractory tumors to standard chemotherapy, targeted therapy, and immunotherapy. On this basis, the rational combination of DUB inhibitors with kinase inhibitors, immune checkpoint blockers, or ferroptosis inducers is emerging as a promising avenue for overcoming therapeutic resistance in oncology, with encouraging prospects for clinical translation.

## 11 Emerging therapeutic strategies: next-generation intervention modalities targeting DUBs

In recent years, therapeutic strategies targeting DUBs have progressively transcended the conventional paradigm of enzymatic activity inhibition, evolving toward the multidimensional regulation of protein stability. Among the emerging approaches, proteolysis-targeting chimeras (PROTACs), deubiquitinase-targeting chimeras (DUBTACs), and molecular glues represent three representative technologies that offer innovative and translationally promising intervention routes by respectively inducing DUB degradation, stabilizing tumor suppressors, and reprogramming DUB substrate specificity.

### 11.1 PROTAC-mediated DUB degradation: functional elimination beyond catalytic inhibition

PROTACs represent a mode of action fundamentally distinct from conventional small-molecule enzyme inhibitors. As bifunctional heterobifunctional molecules, PROTACs simultaneously engage the target protein and an E3 ubiquitin ligase, thereby inducing polyubiquitination of the target and subsequent degradation via the ubiquitin-proteasome system. Taking the USP7-targeting PROTAC molecule U7D-1 as an example, this compound not only suppresses the catalytic activity of USP7 but also induces the complete elimination of the USP7 protein, exerting a more profound impact on downstream signaling pathways than catalytic inhibition alone. Experimental evidence has demonstrated that, compared with the isolated blockade of USP7 catalytic activity, U7D-1-induced USP7 protein degradation produces a more pronounced and sustained antitumor proliferative effect (Pei et al., 2022). The core advantage of the PROTAC strategy lies in its capacity to effectively circumvent several inherent limitations of conventional catalytic inhibitors, including incomplete occupancy of the target protein, residual activity arising from non-enzymatic functions of the target, and functional reactivation resulting from compensatory feedback signaling (Klink et al., 2026). These attributes confer upon PROTAC technology a broad application prospect in the precise intervention of DUBs.

### 11.2 DUBTAC-mediated stabilization of tumor suppressors: a reversed regulatory strategy

In contrast to the directional logic of PROTACs, DUBTACs adopt a functionally complementary strategy. As a class of heterobifunctional molecules, DUBTACs selectively recruit specific deubiquitinating enzymes to the target protein, catalyzing its deubiquitination and thereby enhancing protein stability. The distinctive value of this technology platform resides in its ability to effectively stabilize tumor suppressor proteins that are otherwise rapidly cleared by the UPS under physiological conditions, thus reversing their homeostatic imbalance. To date, researchers have successfully developed a variety of DUBTAC molecules capable of recruiting OTUB1, USP7, USP28, or USP1 to designated substrate proteins, including the mutant p53 as a critical target (Henning et al., 2022). Of particular note, a landmark proof-of-concept study demonstrated that DUBTAC-mediated recruitment of USP7 to the p53 Y220C mutant effectively stabilizes this thermodynamically labile tumor suppressor *in vivo*, achieving substantial tumor regression in preclinical models (Liu J. et al., 2022). This strategy fundamentally subverts the conventional logic of DUB intervention, its core premise is not to inhibit DUB activity, but rather to harness the catalytic function of DUBs for the stabilization of tumor suppressor proteins, thereby inaugurating a novel anticancer paradigm oriented toward protein stability gain (He et al., 2026; Wang et al., 2025).

### 11.3 Molecular glue strategy: reprogramming of substrate selectivity and functional conversion

Molecular glues are small-molecule compounds capable of inducing or stabilizing protein–protein interactions. In the context of DUB intervention, molecular glues can be designed to reshape the substrate recognition profile of USPs, redirecting their substrate preference from oncogenic substrates toward tumor suppressors, thereby effecting a functional reorientation. A molecular glue that simultaneously engages p53 and USP7 has been reported to stabilize p53 by enhancing its deubiquitination, exhibiting explicit antiproliferative activity both in cultured cancer cells and in *in vivo* xenograft models (Li Z. et al., 2024). In addition, bromocriptine has been successfully repurposed as a molecular glue to reinforce the protein–protein interaction between p53 and USP7, resulting in p53 accumulation and suppression of tumor growth. The unique appeal of the molecular glue strategy lies in its independence from DUB activity inhibition; instead, it achieves therapeutic benefit by reprogramming the substrate selectivity of the same enzyme molecule, converting it from an oncogenic driver into a tumor-suppressive effector. This functional conversion mechanism forms a distinct complement to conventional DUB inhibition strategies and offers new avenues for expanding the therapeutic frontiers of DUB-targeted interventions.

## 12 Conclusion and discussion

In summary, the regulatory roles of DUBs in tumors have garnered increasing attention in recent years. Various DUBs exert important and extensive regulatory functions in tumor initiation and progression. Currently, selective inhibitors targeting USP7, USP22, and USP10 are at the preclinical research stage and hold great potential for future application in anti-cancer clinical therapy, particularly as combination agents to overcome chemotherapy resistance and immune resistance in tumor treatment. This review focuses on five DUBs of the USP family, including USP7, USP22, USP10, USP35, and USP4. These enzymes are associated with various cancer types and are correlated with patient prognosis, as summarized in Table 6. They regulate multiple tumor characteristics and predominantly exhibit cancer-promoting effects, making them promising therapeutic targets of current interest. Notably, beyond these five well-studied USP family DUBs, numerous other DUBs from both the USP and OTU families have also been identified as being closely associated with tumor development, as shown in Table 7 (representative rather than exhaustive). Collectively, they exert regulatory functions across different tumor types, various cancer hallmarks, and diverse signaling pathways.

**TABLE 6:** Summary of USP expression and prognostic associations in cancer.

DUB	Cancer types with elevated expression	Prognostic association	Key evidence	References
USP7	Prostate cancer, NSCLC, HCC, neuroblastoma, SCLC, colorectal cancer	↑ overexpression → poor prognosis (prostate: negative correlation with nuclear PTEN)	Overexpression negatively correlated with PTEN nuclear export in prostate cancer samples; USP7 and YAP both upregulated in HCC clinical specimens	(Song et al., 2008)
USP22	Multiple tumor types (breast, NSCLC, prostate, APL, colorectal)	↑ overexpression → poor prognosis (general); ↓ expression → tumor-suppressive in colorectal cancer (context-dependent)	Highly expressed in multiple tumor types; elevated expression associated with poor prognosis in a pan-cancer stem cell signature; tumor-suppressive role in colorectal cancer via mTOR regulation	(Glinsky, 2006)
USP10	Liver cancer (↑), osteosarcoma (↑), colon cancer (↓), renal clear cell carcinoma (↓)	Dual: ↑ in liver cancer → poor; ↓ in ccRCC → tumor-suppressive; ↓ USP10/SIRT6 in colon cancer	Positively correlated with YAP in liver cancer, tumor-promoting role validated in animal models; significantly downregulated in ccRCC	(Yuan et al., 2010; Zhu et al., 2020)
USP35	Gastric cancer, thyroid cancer, colorectal cancer, ER+ breast cancer, ovarian cancer, lung cancer	↑ overexpression → poor prognosis (general tumor-promoting role)	Significantly overexpressed in nine cancer types by pan-cancer bioinformatics analyses; highly expressed in human lung cancer tissues	(Yan et al., 2025b)
USP4	Breast cancer, gastric cancer, glioblastoma	↑ overexpression → poor prognosis (breast: PAK5-USP4 axis validated in clinical samples)	PAK5 and USP4 markedly elevated in clinical breast cancer samples; high expression of both proteins associated with poorer survival	(Wang et al., 2020b)

NSCLC, Non-small cell lung cancer; HCC, hepatocellular carcinoma; SCLC, small cell lung cancer; APL, acute promyelocytic leukemia; ccRCC, clear cell renal cell carcinoma; ER, estrogen receptor.

**TABLE 7:** Additional representative cancer-associated deubiquitinases and their stabilized substrates implicated in tumorigenesis.

DUB	Representative substrates	Key cancer hallmarks	Representative cancer types	References
USP1	FANCD2, PCNA, ID2	DNA repair, proliferation	BRCA-mutant/HRD-deficient solid tumors	(Nijman et al., 2005; Cadzow et al., 2024)
USP14	Ubiquitinated proteasome substrates (global)	Protein homeostasis, anti-apoptosis	Multiple myeloma, endometrial	(Vogel et al., 2016; Wang et al., 2016)
USP8	EGFR, PD-L1, Frizzled	Proliferation, immune evasion, Wnt signaling	NSCLC, cervical	(Ke et al., 2025; Xiong et al., 2022)
USP9X	Mcl-1, SMAD4, β-catenin, Survivin	Anti-apoptosis, proliferation	Multiple myeloma, pancreatic, breast	(Schwickart et al., 2010; Dupont et al., 2009)
USP13	Mcl-1, PTEN, STAT1	Anti-apoptosis, immune signaling	Cervical, ovarian, glioblastoma	(Morgan et al., 2021; Zhang et al., 2013)
USP28	c-Myc, c-Jun, LSD1, NICD1	Proliferation, stemness	Colorectal, breast, NSCLC	(Popov et al., 2007; Diefenbacher et al., 2014)
USP47	β-catenin, YAP, Snail	Proliferation, EMT/metastasis	Colorectal, gastric	(Pan et al., 2020; Shi et al., 2015)
USP21	FOXM1, BRCA2, MEK2, ZEB1, G3BP1, PD-L1, EZH2, β-catenin, HIF1-α, YAP (via YOD1)	Proliferation, DNA repair, EMT, immune evasion, hypoxia response, Wnt/Hippo signaling	Breast (BLBC), colorectal, esophageal SCC, cholangio-carcinoma, pancreatic, prostate, HCC, glioblastoma	(Arceci et al., 2019; Liu et al., 2017; Li et al., 2018; Lin and Lu, 2024; Guo et al., 2024)
USP27X	Snail1, Cyclin D1 (CCND1), Bim (BCL2L11), cFLIP_L, CBX2	EMT/metastasis, proliferation, apoptosis regulation (dual role), tumor suppression via Bim/cFLIP	Breast, pancreatic, melanoma, NSCLC	(Lambies et al., 2019; Alam et al., 2022; Weber et al., 2016; Dold et al., 2022; Xing et al., 2023)
USP51	ZEB1, TWIST1, PD-L1, HIF1A, GRP78, NEK8, GPX4 (promotes degradation)	EMT/metastasis, stemness, immune evasion, hypoxia response, chemotherapy resistance	NSCLC, breast (TNBC), colorectal, esophageal SCC	(Zhang et al., 2020; Li et al., 2023; Mu et al., 2023; Chen et al., 2023a; Ou et al., 2025)

This list is representative rather than exhaustive. BLBC, Basal-like breast cancer; SCC, squamous cell carcinoma; TNBC, Triple-negative breast cancer.

Considerable progress has been made in identifying selective small-molecule inhibitors of DUBs. As of mid-2026, the clinical translation of specific DUB inhibitors remains in early stages (Table 8). The only DUB inhibitor to have completed a clinical trial to date is KSQ-4279 (RO7623066), a first-in-class allosteric USP1 inhibitor that demonstrated acceptable safety and PK/PD activity in a Phase 1 dose-escalation study in patients with advanced solid tumors (NCT05240898) (Torrado et al., 2025). Additional USP1 inhibitors currently under clinical evaluation include XL309/ISM3091 (NCT05932862) and HSK39775 (NCT06314373), with SIM0501 (NCT06331559) and TNG348 (NCT06065059) in terminated status. Separately, VLX1570, a USP14/UCHL5 inhibitor, entered a Phase 1 trial in relapsed/refractory multiple myeloma but was terminated due to severe pulmonary toxicity observed at the 1.2 mg/kg dose level (NCT02372240) (Rowinsky et al., 2020). None of the USP7-, USP22-, USP10-, USP35-, or USP4-targeted inhibitors have advanced beyond preclinical evaluation. These data indicate that the clinical development of DUB-targeted agents has progressed beyond the preclinical stage, although significant challenges in selectivity, therapeutic window, and target-specific toxicity remain.

**TABLE 8:** Clinical-stage specific deubiquitinase inhibitors.

DUB target	Compound	Clinical phase	Status	Indication	ClinicalTrials.gov ID
USP1	RO7623066 (KSQ-4279)	Phase 1	Completed	Advanced solid tumors	NCT05240898
USP1	XL309 (ISM3091)	Phase 1	Recruiting	Advanced solid tumors	NCT05932862
USP1	SIM0501	Phase 1	Terminated	Advanced solid tumors	NCT06331559
USP1	TNG348	Phase 1/2	Terminated	BRCA1/2-mutant or HRD+ solid tumors	NCT06065059
USP1	HSK39775	Phase 1/2	Recruiting	Advanced solid tumors	NCT06314373
USP14/UCHL5	VLX1570	Phase 1/2	Terminated	Relapsed/refractory multiple myeloma	NCT02372240

However, the tumor treatment targeting DUBs also faces certain challenges and difficulties. On the one hand, ubiquitin signaling and ubiquitin modifications are widespread and complex within cells, and a given deubiquitinating enzyme typically regulates a diverse array of substrates and functions. Therefore, targeting a specific DUB may lead to alterations in multiple downstream pathways, potentially resulting in poor specificity or increased toxic side effects. On the other hand, the diversity of substrates regulated by DUBs implies that a single DUB might simultaneously exert different, even opposing effects. For example, as discussed above, USP7 can directly deubiquitinate and stabilize p53, or alternatively deubiquitinate and stabilize the corresponding E3 ubiquitin ligase Mdm2, thereby promoting p53 degradation. Accumulating evidence indicates that the role of a particular DUB in cancer is often highly context-dependent. In one type of cancer or under certain conditions, it may promote cancer progression, whereas in another cancer type or context, it may exert the opposite, tumor-suppressive effect. This underscores that clinical applications in tumor therapy require more robust biological research evidence and careful consideration. When confronting the difficulties in cancer treatment, it is often necessary to focus on the major regulatory pathways and primary effects, while also paying attention to the potential influences arising from other pathways and secondary effects. Moreover, nearly 100 DUBs exist in cells, some of which may have redundant regulatory functions with respect to both substrates and functional outcomes. Consequently, the specific inhibitory effect of a given DUB might be compensated by the activity of other DUBs within the cell, an issue that requires further comprehensive biological investigation for clarification.

Despite the aforementioned problems and challenges, based on current research progress, multiple DUBs still hold great promise as targets for tumor therapy. Targeted cancer therapies directed at DUBs are currently progressing vigorously. Several drug candidates under development have a strong chance of advancing to the clinical research stage in the near future. Future research in this field can be mainly divided into two major directions. On the one hand, focusing on the currently reported potential targets for tumor therapy, efforts are being made to develop highly efficient and selective inhibitors and other forms of therapeutic agents, which, after passing through preclinical and clinical research, may be applied in future tumor clinical treatment. On the other hand, further in-depth investigations are needed to address various scientific issues concerning the catalytic mechanisms, regulatory substrates, signaling pathways, and regulatory functions of DUBs, thereby laying the foundation for future DUB-targeted clinical cancer therapy. In addition, the development of selective inhibitors can also serve as a research tool to further advance our understanding of the regulatory functions and mechanisms of DUBs.
